# A New Prognostic Parameter Associated With Recurrence in Patients With Nasopharyngeal Cancer Treated With Chemoradiotherapy: The Ratio of the Hemoglobin-to-Red Cell Distribution Width

**DOI:** 10.7759/cureus.39907

**Published:** 2023-06-03

**Authors:** Yakup Bozkaya, Muhammet Dilber, Ahmet M Bilgili, Caner Aktaş

**Affiliations:** 1 Medical Oncology, Yeni Yüzyıl University, İstanbul, TUR; 2 Otolaryngology-Head and Neck Surgery, Dilber ENT and Aesthetic Clinic, İstanbul, TUR; 3 Otolaryngology-Head and Neck Surgery, Cyprus International University, Faculty of Medicine, Lefkoşa, CYP; 4 Clinic of Radiation Oncology, University of Health and Sciences Turkey, Istanbul Training and Research Hospital, İstanbul, TUR

**Keywords:** prognostic factor, red cell distribution width, hemoglobin, chemo-radiotherapy, locally advanced nasopharyngeal cancer

## Abstract

Introduction: This study aims to investigate the prognostic significance of the pre-treatment hemoglobin-red blood cell distribution width (RDW) ratio (HRR) in terms of overall survival (OS) and disease-free survival (DFS) in patients with locally advanced nasopharyngeal cancer (LANC) treated with chemoradiotherapy.

Methods: Patients with LANC who attended the oncology clinic between October 2010 and June 2020 were retrospectively screened. HRR was calculated as hemoglobin (g/dL) divided by the RDW (%). Patients were assigned to either the low HRR group or the high HRR group.

Results: A total of 102 patients were included in the study. The cut-off value for HRR was taken as 0.97. Between the low and high HRR groups, mean age, Eastern Cooperative Oncology Group (ECOG) performance score, gamma-glutamyl transferase (GGT), albumin and lactate dehydrogenase (LDH) levels, weight loss at diagnosis, and recurrence and metastasis rate were significantly different. In the low HRR group, OS and DFS were 44.4 (95% CI: 4.9-83.8) and 15.7 months (95% CI: 0.1-36.2), respectively, but could not be reached in the high HRR group (p<0.001). In the multivariate analysis, low HRR was shown to be an independent factor in terms of both OS (p=0.004, hazard ratio (HR)=3.07, 95% CI: 1.444-6.529) and DFS (p<0.001, HR=3.94, 95% CI: 1.883-8.244).

Conclusion: This is the first study showing that HRR is an independent prognostic marker for OS and DFS in patients with LANC treated with chemoradiotherapy. Thus, HRR can be used as an easily applicable, inexpensive marker in clinical practice in this patient group.

## Introduction

Nasopharyngeal cancer differs from other head and neck cancers because of its clinical behavior, treatment, relationship with Epstein-Barr virus (EBV) infection, and geographical distribution. Early-stage patients are treated with radiotherapy alone, while advanced-stage patients require a combination of chemoradiotherapy [1]. In some studies, concomitant chemoradiotherapy has been shown to be superior to radiotherapy alone or sequential therapy in terms of locoregional control [2]. Despite combined chemoradiotherapy, some patients develop disease recurrence or metastasis within a few years. Therefore, the identification of a prognostic factor indicating early progression will allow a better therapeutic approach to patients with locally advanced nasopharyngeal cancer (LANC).

The American Joint Committee on Cancer (AJCC) tumor node and metastasis (TNM) classification continues to be used as the most common tool for predicting prognosis and designing treatment options [3]. The TNM staging system is far from perfect as it only considers tumor behavior without considering prognostic factors such as clinicopathological data and tumor-associated markers. Thus, it will come as no surprise that patients with the same TNM staging show heterogeneous survival outcomes. Therefore, it will be important to define accurate prognostic indicators to support TNM staging.

Recent studies have shown that Epstein-Barr virus DNA load, epidermal growth factor receptor overexpression, and various inflammatory-based prognostic scores (prognostic nutrition index, c-reactive protein, neutrophil-to-lymphocyte ratio (NLR), platelet-to-lymphocyte ratio (PLR)) are associated with recurrence, metastasis, and prognosis in patients with nasopharyngeal cancer [4-7]. However, some of these markers are not routinely applied in clinical practice due to their high cost and large variability during experiment time. Therefore, it would be appropriate to use inexpensive and applicable prognostic markers that are easy to access. Complete blood count (CBC) is an easily applicable test that can be performed on cancer patients in routine practice. The hemoglobin/red blood cell distribution width (RDW) ratio (HRR) measured in a CBC has been shown to be prognostic in various cancers [8-13]. In addition to its prognostic value, HRR was found to be a valuable or helpful indicator in the diagnosis of nasopharyngeal cancer in a study involving patients with nasopharyngeal cancer [14]. Based on these results, our aim in this study is to assess the prognostic effect of HRR on overall survival (OS) and disease-free survival (DFS) in LANC patients treated with chemoradiotherapy.

## Materials and methods

Patients with nasopharyngeal cancer who attended the oncology clinic of our hospital between October 2010 and June 2020 were retrospectively screened. Patients with nasopharyngeal cancer histopathologically proven by biopsy were included in the study. All patients were stage II-IVA disease (the American Joint Committee on Cancer (AJCC) staging 8th edition) receiving definitive chemoradiotherapy. Other eligibility criteria for the study were an Eastern Cooperative Oncology Group (ECOG) performance score of 0-2, patients with adequate renal (glomerular filtration rate >60), cardiac and liver function, over 18 years of age and without a previous diagnosis of cancer (excluded basal cell carcinoma (BCC)). Patients with any active immunological, hematological and infection diagnosis, distant metastases, incomplete follow-up, and/or receiving only one of the chemotherapy or only radiotherapy were not included in the study.

Clinicopathological characteristics such as age, gender, smoking status, pathological subtype, comorbidity, ECOG performance score, weight loss (which was defined as a loss of more than 5% of average body weight over six months), and laboratory data including biochemical and complete blood count parameters were examined from the patient files. In addition, it was also evaluated in terms of the chemotherapeutic agent received during radiotherapy with consolidation chemotherapy. A complete blood count was obtained at the time of diagnosis as a measure of routine clinical practice. HRR was calculated using the following formula: Hb (g/dL) divided by RDW (%). According to the receiver operating characteristic (ROC) analysis, the cut-off point for HRR was 0.97 (95% CI: 0.75-0.91). Per this cut-off value, patients were divided into two groups: low and high HRR.

As a radiotherapy technique, the 2/3D conformal technique was applied before 2012, and the intensity-modulated radiotherapy (IMRT) technique was applied after 2012. The standard dose for the 2/3D conformal technique was 70 Gy to the primary area and 50-60 Gy to the involved lymph nodes in 35-day fractions. The dose and fractions for IMRT were 70 Gy/2.12 Gy per fraction for high-risk target volumes (primary tumor volume and involved nodes), 60 Gy/1.8 Gy per fraction for intermediate-risk, and 54 Gy/1.65 Gy per fraction for low-risk target volumes. All patients were administered either 40 mg/m^2^ of cisplatin once a week or 100 mg/m^2^ of cisplatin every three weeks as an intravenous infusion starting from the first day of radiotherapy. Cisplatin plus 5-fluorouracil was administered in two to four cycles every three weeks as consolidation chemotherapy. Patients with a complete response after treatment were followed up every three months for the first two years, then the follow-up interval was extended.

Statistical analysis

All statistical analyses were performed using the SPSS 22 Windows version (IBM Corp., Armonk, NY). A p-value of <0.05 was considered statistically significant. The variables were investigated using visual (histograms, probability graphs) and analytical methods (Kolmogorov-Smirnov and Shapiro-Wilk tests) to determine whether they were normally distributed. The comparison of clinicopathological features between the groups was made using the chi-square or Fisher's exact test. The ideal cut-off values for HRR, neutrophil/lymphocyte ratio (NLR), and platelet/lymphocyte ratio (PLR) were determined by ROC curve analysis. Survival analysis of the patients was performed using the Kaplan-Meier method, and the log-rank test was used to compare subgroups. Possible factors determined by univariate analysis were put into Cox regression analysis (with backward selection) to determine independent markers for OS and DFS. Hazard ratios (HRs) were presented in a 95% confidence interval with two-sided p-values. OS is defined as the time from the date of diagnosis to the date of the last follow-up or death from any cause. DFS was measured from day one of treatment until the day of locoregional recurrence and metastasis, or the last follow-up.

## Results

The demographic characteristics of the patients are summarized in Table 1. A total of 102 patients with nasopharyngeal cancer, 88 (86.3%) male and 14 (13.7%) female, were included in the study. The mean age was 49.9 (range: 18-80, standard deviation (SD): 13.9). At the time of diagnosis, 92 patients (90.2%) had an ECOG performance score of 0-1, and 26 patients (25.5%) had weight loss. Based on the AJCC grading standards, 76 patients (74.5%) were at stages II-III, and 26 patients (25.5%) were at stage IVA. The median hemoglobin level in the entire patient group was 14.0 g/dL (range: 8.6-16.7 g/dL), and 15 patients were evaluated for anemia (<11 g/dL for females and 12 g/dL for males). The median RDW was 13.9 (%) (range: 11.9-19.1%).

**Table 1:** Demographic characteristics of the patients. ECOG: Eastern Cooperative Oncology Group; HRR: hemoglobin/red cell distribution width ratio; HR: hazard ratio; CI: confidence interval; NLR: neutrophil/lymphocyte ratio; PLR: platelet/lymphocyte ratio; TNM: tumor node and metastasis; RT: radiotherapy; IMRT: intensity-modulated radiotherapy.

Characteristics	Low HRR (<0.97)	High HRR (≥0.97)	Total	P-value
	N=31 (%)	N=71 (%)	N=102(%)	
Age (mean, years)	56.3	47.1	49.9	0.002
Gender				0.08
Female	7 (22.5)	7 (9.8)	14 (13.7)	
Male	24 (77.5)	64 (90.2)	88 (86.3)	
Smoking				0.67
Yes	17 (54.8)	35 (49.3)	52 (50.9)	
No	14 (45.2)	36 (50.7)	50 (49.1)	
Alcohol				
Yes	4 (12.9)	15 (21.1)	19 (18.6)	0.32
No	27 (87.1)	56 (79.9)	83 (81.4)	
ECOG				
0-1	24 (77.5)	68 (95.8)	92 (90.2)	0.004
2	7 (22.5)	3 (4.2)	10 (9.8)	
Weight loss				
Yes	13 (41.9)	13 (18.3)	26 (25.4)	0.01
No	18 (58.1)	58 (81.7)	76 (74.6)	
Comorbidity				
Yes	11 (35.4)	13 (18.3)	24 (23.5)	0.06
No	20 (64.6)	58 (81.7)	78 (76.5)	
TNM				
II-III	20 (64.6)	56 (79.9)	76 (74.6)	0.12
IVA	11 (35.4)	15 (21.1)	26 (25.4)	
Chemotherapy				
Weekly	24 (77.5)	64 (90.2)	88 (86.3)	0.08
Three-weekly	7 (22.5)	7 (9.8)	14 (13.7)	
GGT				
Normal	17 (54.8)	64 (90.2)	81 (79.4)	<0.001
High	14 (45.2)	7 (9.8)	21 (20.6)	
LDH				
Normal	19 (61.2)	65 (91.5)	84 (82.4)	<0.001
High	12 (38.8)	6 (8.5)	18 (17.6)	
Hemoglobin (gm/dL)				
≥10	25 (80.6)	71 (100)	96 (94.1)	<0.001
<10	6 (19.4)	-	6 (5.9)	
Albumin (g/L)				
<45	28 (90.3)	51 (71.8)	79 (77.5)	0.04
≥45	3 (9.7)	20 (28.2)	23 (22.5)	
NLR (median)	2.83	2.77	2.79	0.42
PLR (median)	178	147	150	0.49
Consolidation chemotherapy				
Yes	4 (12.9)	15 (21.1)	19 (18.6)	0.32
No	27 (87.1)	56 (78.9)	83 (81.4)	
Weekly/three-weekly cisplatin				
Delay/interrupted	9 (29)	12 (16.9)	21 (20.6)	0.16
Completed	22 (71)	59 (83.1)	81 (79.4)	
RT technique				
2D/3D RT	7 (22.5)	14 (19.7)	21 (20.5)	0.74
IMRT	24 (77.5)	57 (80.3)	81 (79.5)	
Recurrence/metastasis	25 (80.6)	13 (18.3)	38 (37.3)	<0.001

Eighty-one patients (79.4%) were treated with the IMRT technique, while 21 (20.6%) patients were treated with the 2/3D conformal RT technique. All patients received cisplatin as a chemotherapeutic agent with radiotherapy except for two patients. Eighty-six patients (86%) and 14 patients (14%) received weekly and three-week cisplatin chemotherapy, respectively. Nineteen patients (18.6%) received two to four cycles of the cisplatin/fluorouracil chemotherapy regimen after chemoradiotherapy. During radiotherapy, 81 patients (79.4%) completed chemotherapy treatment, while the remaining patients had to interrupt (19 patients) or discontinue (2 patients) therapy due to toxicity and other reasons. A total of 38 patients (37.3%) experienced recurrence or metastasis, and 24 patients received first-line chemotherapy (the most commonly used regimen was gemcitabine/cisplatin in 15 patients). Thirty-three patients (32.3%) died of nasopharyngeal cancer during follow-up.

According to the ROC curve, 31 and 71 patients were included in the low and high HRR groups, respectively. There were statistically significant differences between the two groups regarding clinical and laboratory characteristics. In the low HRR group, mean age (p=0.002), ECOG performance score 2 (p=0.004), weight loss at diagnosis (p=0.01), gamma-glutamyl transferase level (GGT) (p<0.001), lactate dehydrogenase (LDH) level (p<0.001), and recurrence and metastasis (p<0.001) were found to be higher. However, hemoglobin (p<0.001) and albumin (p=0.04) levels were higher in the high HRR group.

**Figure 1:**
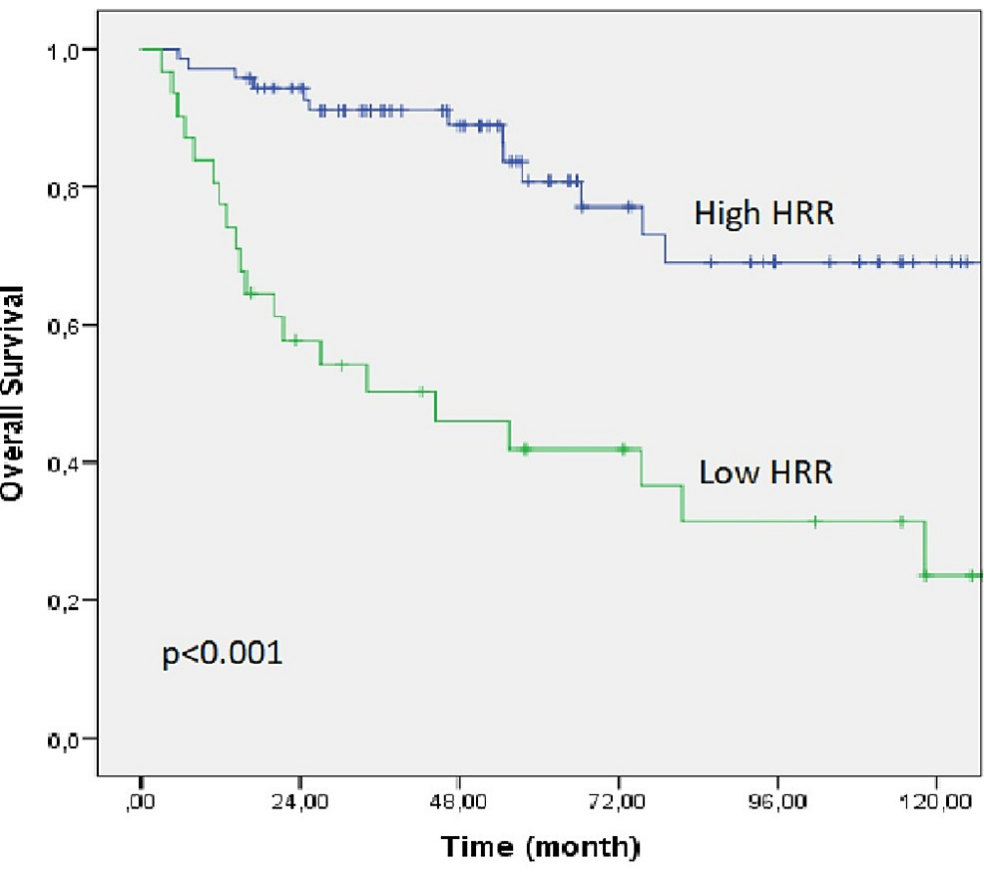
Kaplan-Meier curve for overall survival of the low HRR and high HRR groups. HRR: hemoglobin/red cell distribution width ratio.

**Figure 2:**
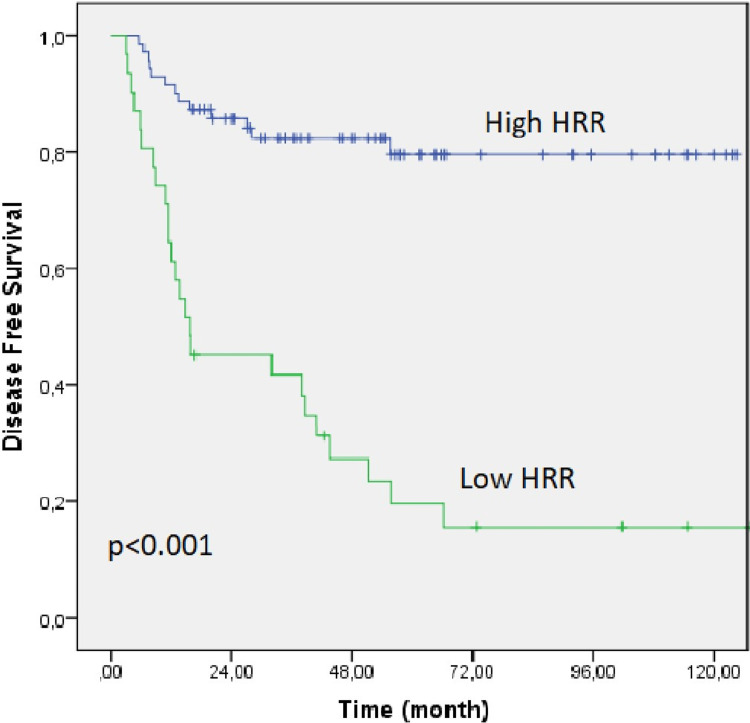
Kaplan-Meier curve for disease-free survival of the low HRR and high HRR groups. HRR: hemoglobin/red cell distribution width ratio.

The median follow-up in the entire group was 48.2 months (range: 3.1-131.3 months), and the median OS and DFS were not reached. In the low HRR group, OS and DFS were 44.4 (95% CI: 4.9-83.8) and 15.7 months (95% CI: 0.1-36.2), respectively, whereas, in the high HRR group, they were not achieved. The Kaplan-Meier OS and DFS curves of both groups are shown in Figures 1, 2.

The factors affecting DFS in the entire patient group were evaluated in the univariate analysis. Low HRR (p<0.001), ECOG performance score of 2 (p<0.001), male gender (p=0.05), stage IVA disease (p<0.001), weight loss at diagnosis (p<0.001), high GGT (p=0.007), high LDH (p=0.01), interruption or discontinuation of weekly/three-weekly cisplatin treatment (p=0.003) were significant factors. Multivariate analysis was performed among the factors found significant in the univariate analysis. Weight loss at diagnosis (p<0.001), stage IVA disease (p<0.001), low HRR (p<0.001), and high GGT (p=0.02) were significant prognostic indicators for shorter DFS (Table 2).

**Table 2:** Univariate and multivariate analysis for disease-free survival. CI: confidence interval; ECOG: Eastern Cooperative Oncology Group; GGT: gamma-glutamyl transferase; IMRT: intensity-modulated radiotherapy; LDH: lactate dehydrogenase; HRR: hemoglobin/red cell distribution width ratio; HR: hazard ratio; NLR: neutrophil/lymphocyte ratio; RT: radiotherapy; PLR: platelet/lymphocyte ratio; TNM: tumor node and metastasis.

	Univariate analysis			Multivariate analysis		
	HR	95% CI	p-value	HR	95% CI	p-value
Age, years						
<50	1.70	0.932-3.432	0.08			
≥50						
Gender						
Female	2.17	0.991-4.753	0.05	1.30	0.539-3.136	0.56
Male						
ECOG						
1	4.45	2.098-9.453	<0.0001	1.20	0.480-3.031	0.68
2						
TNM						
III	4.26	2.242-8.123	<0.0001	6.19	2.935-12.868	<0.001
IVA						
Weight loss						
Yes	5.89	3.030-11.450	<0.0001	3.90	1.890-8.067	<0.001
No						
HRR						
Low HRR	6.32	3.227-12.408	<0.0001	3.94	1.883-8.244	<0.001
High HRR						
NLR						
<2.75	1.22	0.646-2.316	0.53			
≥2.75						
PLR						
<149	1.10	0.584-2.099	0.75			
≥149						
GGT						
Normal	2.89	1.337-6.267	0.007	2.33	1.095-4.959	0.02
High						
LDH						
Normal	2.64	1.177-5.939	0.01	1.65	0.605-4.534	0.32
High						
RT technique						
2D/3D RT	1.13	0.749-1.710	0.55			
IMRT						
Chemotherapy						
Weekly	1.05	0.592-1.530	0.83			
Three-Weekly						
Weekly/three-weekly cisplatin						
Delay/interrupted	1.70	1.204-2.406	0.003	1.21	0.843-1.744	0.30
Completed						

The factors affecting OS in the entire patient group were evaluated in the univariate and multivariate analyses (Table 3). Low HRR (p<0.001), ≥50 years of age (p=0.005), ECOG performance score of 2 (p<0.001), stage IVA disease (p=0.01), weight loss at diagnosis (p<0.001), and interruption or discontinuation of weekly/three-weekly cisplatin treatment (p<0.001) were significant factors. In the multivariate analysis, stage IVA disease (p=0.02), weight loss at diagnosis (p=0.01), low HRR (p=0.004), and interruption or discontinuation of weekly/three-weekly cisplatin treatment (p<0.001) were found to be associated with shorter OS (Table 3).

**Table 3:** Univariate and multivariate analysis for overall survival. CI: confidence interval; ECOG: Eastern Cooperative Oncology Group; GGT: gamma-glutamyl transferase; IMRT: intensity-modulated radiotherapy; LDH: lactate dehydrogenase; HRR: hemoglobin/red cell distribution width ratio; HR: hazard ratio; NLR: neutrophil/lymphocyte ratio; RT: radiotherapy; PLR: platelet/lymphocyte ratio; TNM: tumor node and metastasis.

	Univariate analysis			Multivariate analysis		
	HR	95% CI	p-value	HR	95% CI	p-value
Age, years						
<50	2.91	1.383-6.163	0.005	1.42	0.605-3.370	0.41
≥50						
Gender						
Female	1.89	0.814-4.432	0.13			
Male						
ECOG						
0-1	1.20	0.461-3.122	0.71			
2						
TNM						
III	2.34	1.156-4.765	0.01	2.18	1.087-4.397	0.02
IVA						
Weight loss						
Yes	3.96	1.868-8.422	<0.0001	2.46	1.169-5.190	0.01
No						
HRR						
Low HRR	4.57	2.257-9.259	<0.0001	3.07	1.444-6.529	0.004
High HRR						
NLR						
<2.75	1.11	0.528-2.360	0.77			
≥2.75						
PLR						
<149	1.49	0.703-3.168	0.29			
≥149						
GGT						
Normal	2.15	0.793-5.874	0.13			
High						
LDH						
Normal	2.06	0.689-6.208	0.19			
High						
RT technique						
2D/3D RT	1.8	0.776-3.016	0.28			
IMRT						
Chemotherapy						
Weekly	1.03	0.559-1.900	0.92			
Three-weekly						
Weekly/three-weekly cisplatin						
Delay/interrupted	2.13	1.447-3.149	<0.001	4.63	2.024-10.591	<0.001
Completed						

## Discussion

Patients diagnosed with nasopharyngeal cancer are highly sensitive to chemoradiotherapy, but some patients may develop recurrence or metastasis after curative treatment, and survival may be poor, especially in these patients. Determining prognostic factors that may positively predict the outcome of treatment will help identify patients who may benefit from more intensive multimodality treatment combinations and improve survival outcomes. Therefore, it is of great interest to determine the prognostic factors that could potentially predict the outcome. In our study involving patients with LANC treated with chemoradiotherapy for this purpose, patients with low HRR (compared to high HRR), according to ROC analysis, had shorter OS (p=0.004, hazard ratio (HR)=3.07, 95% CI: 1.444-6.529). Moreover, in the multivariate analysis, low HRR was an independent prognostic factor for shorter DFS (p<0.0001, HR=3.94, 95% CI: 1.883-8.244). Thus, more intensive or new treatment regimen studies may be needed to achieve better survival in patients with nasopharynx who will receive chemoradiotherapy with low HRR during the diagnosis period. 

Red cell distribution width is a widely used parameter found in the complete blood count that determines anisocytosis in erythrocytes in the blood. It has been shown that an increase in RDW is triggered by nutritional deficiency caused by iron, vitamin B12, and folic acid deficiency and is associated with chronic inflammation and acute phase inflammation markers, such as C-reactive protein, tumor necrosis factor (TNF), and interleukin-6 [15,16]. It has been reported that RDW has a prognostic relationship in cardiovascular, pulmonary and hepatic diseases, and it has subsequently been shown to be a poor prognostic factor in various malignant diseases, especially in the lung and breast [17-20]. In a meta-analysis of 49 studies involving 19,790 patients with many cancer types, especially breast, lung, stomach, colon, liver, and larynx cancers. Wang et al. reported that high RDW levels were associated with shorter OS, DFS, progression-free survival (PFS), and recurrence-free survival (RFS) [21]. The prognostic value of RDW in head and neck cancers has been shown primarily in patients with laryngeal cancer. In the study of Kara et al., it was shown that high RDW was associated with mortality in 81 patients with laryngeal cancer [22]. In another study involving patients with laryngeal cancer, Bozkurt et al. showed that high RDW was associated with a higher risk of local recurrence in patients with operated laryngeal cancer [23]. Although studies on the prognostic value of RDW in patients with nasopharynx cancer are limited. Wang et al. published a study on the combination of red blood cell distribution width and body mass index (COR-BMI) prognostic index, which consists of the combination of RDW and body mass index (BMI) in patients with nasopharynx cancer. In this study, while COR-BMI had a prognostic value for OS (HR for COR-BMI 1: 1.239, 95% CI: 1.012-1.590; HR for COR-BMI 2: 2.367, 95% CI: 1.311-4.274, p= 0.013), it was not for DFS (P=0.482). Although it was a study with a large number of patients (n=2318), it included a heterogeneous patient group (metastatic and non-metastatic) [24].

Tumor hypoxia is a surrogate marker for resistance to treatment in patients receiving chemoradiotherapy. Anemia is considered an indirect indicator of tumor hypoxia [25]. There are several studies investigating the prognostic factors associated with hypoxia in patients with nasopharyngeal cancer. Zhang et al. reported that pre-treatment anemia (hemoglobin <12 g/L in males and 11 g/L in females) was associated with inferior DFS (HR=2.15, 95 %CI 1.62-2.85, p<0.001) and distant metastasis-free survival (DMFS) (HR=1.53, 95 % CI 1.08-2.17, P=0.018) in 5830 patients with stage I-IV nasopharyngeal cancer [26]. In another study conducted in nasopharyngeal cancer patients treated with only IMRT, a lower pre-treatment hemoglobin level was associated with decreased DMFS (P=0.007, HR=2.555, 95% CI: 1.294-5.046). Gao et al. showed that 520 nasopharyngeal cancer patients treated with definitive radiotherapy (three-dimensional radiotherapy) did not differ in five-year locoregional recurrence-free survival (p=0.337) and OS (p=0.299) in patients with and without pre-treatment anemia (hemoglobin level <13 g/L in men and <12 g/L in women). However, the change in hemoglobin levels during radiotherapy was an important factor affecting overall survival [27]. The different results of the prognostic effect of anemia in these studies of nasopharyngeal patients can be considered as the low number of anemic patients in the studies, probably because the general condition of nasopharyngeal patients is good, and therefore they are rarely anemic before treatment. In addition, it is quite remarkable that there is no standard cut-off value for low hemoglobin levels in the studies mentioned above.

In 2016, Sun et al. first showed that HRR is of prognostic value for OS in patients with esophageal cancer receiving curative treatment [9]. In another study involving 205 patients with head and neck cancer, low HRR was shown to have prognostic significance for event-free survival (EFS), not OS [10]. However, this study included a heterogeneous patient group (oropharynx, oral cavity, nasopharynx, larynx, salivary gland, paranasal sinus, etc.), and there were only two nasopharynx patients. Subsequently, low HRR was shown to be a prognostic factor for poor OS and PFS in patients with advanced-stage non-small cell lung cancer treated with chemotherapy [8]. After the results of the above studies, the prognostic importance of HRR in various cancer types, such as kidney, stomach, small cell lung cancer, and bladder cancer, has been demonstrated [11-13,28]. The only study on HRR in patients with nasopharynx was conducted by Lin et al., and it was reported that HRR is a valuable indicator for the diagnosis of patients with nasopharyngeal cancer [14]. Although the prognostic value of both hemoglobin and RDW has been reported separately in patients with nasopharyngeal cancer, the prognostic effect of HRR is not clear. As far as we know, it was demonstrated for the first time in our study that included patients with LANC treated with chemoradiotherapy. Perhaps this result was not surprising due to the demonstration of the prognostic effect of both hemoglobin and RDW in patients with different cancers. However, our study was important because it included only a specific disease subgroup and patients receiving chemoradiotherapy, and it was an independent prognostic indicator for DFS in these patients. In addition, HRR is much more likely to provide a more objective assessment since hemoglobin and RDW are affected by many causes other than cancer.

Several studies are showing that low HRR is associated with poor prognostic factors. In the study conducted on patients with lung, esophageal and gastric cancer, patients with low HRR were associated with prognostic factors such as a more advanced stage, more lymph node involvement, and weight loss [8,9,12]. In our study, patients with low HRR had a higher ECOG performance score of 2, weight loss at the time of diagnosis, and recurrence or metastasis compared to the high HRR group. Thus, low HRR may be an indicator of aggressive tumor behavior. These findings support previous studies showing that low hemoglobin and a high RDW value are closely related to aggressive tumor behavior [29,30].

Although our study was the first to show that HRR was an independent prognostic indicator for OS and DFS in patients receiving chemoradiotherapy with LANC, it had several limitations. The first was that it had a single-center, retrospective design, and a limited number of patients. The other was impossible to exclude inflammatory/inflammatory and autoimmune diseases that may affect HRR levels. In addition, another point to be considered was that we did not know the results of parameters that are not routinely checked in practice, such as iron, vitamin B12, and folic acid, which may affect the HRR level. The lack of a standard cut-off value is another limitation in these studies and our study since each study determines the cut-off value with different statistical methods. Therefore, it would be appropriate to conduct large prospective studies using a standard uniform statistical method, considering these parameters and diseases that may affect the HRR level.

## Conclusions

As a result, although patients with nasopharyngeal cancer are sensitive to chemoradiotherapy, recurrence and metastasis may develop in some patients. It will be important for the results to determine various prognostic and predictive parameters in order to predict these patients and adapt the treatment accordingly. This is the first study to investigate the prognostic value of HRR in patients receiving chemoradiotherapy with LANC, and it was found to be an independent prognostic parameter for both DFS and OS. Thus, HRR can be used as an easily applicable inexpensive marker in clinical practice in this patient group.
